# Supplementary material for: Selective binding of the PHD6 finger of MLL4 to histone H4K16ac links MLL4 and MOF
Source: Nat Commun. 2019 May 24;10:2314. doi: 10.1038/s41467-019-10324-8 (PMC6534582; doi:10.1038/s41467-019-10324-8)
Supplement: Supplementary file 1 — Supplementary Information [file 41467_2019_10324_MOESM1_ESM.pdf]

Supplementary Information

Selective binding of the PHD6 finger of MLL4 to histone H4K16ac links MLL4 and MOF

Yi Zhang, Younghoon Jang, et al.,

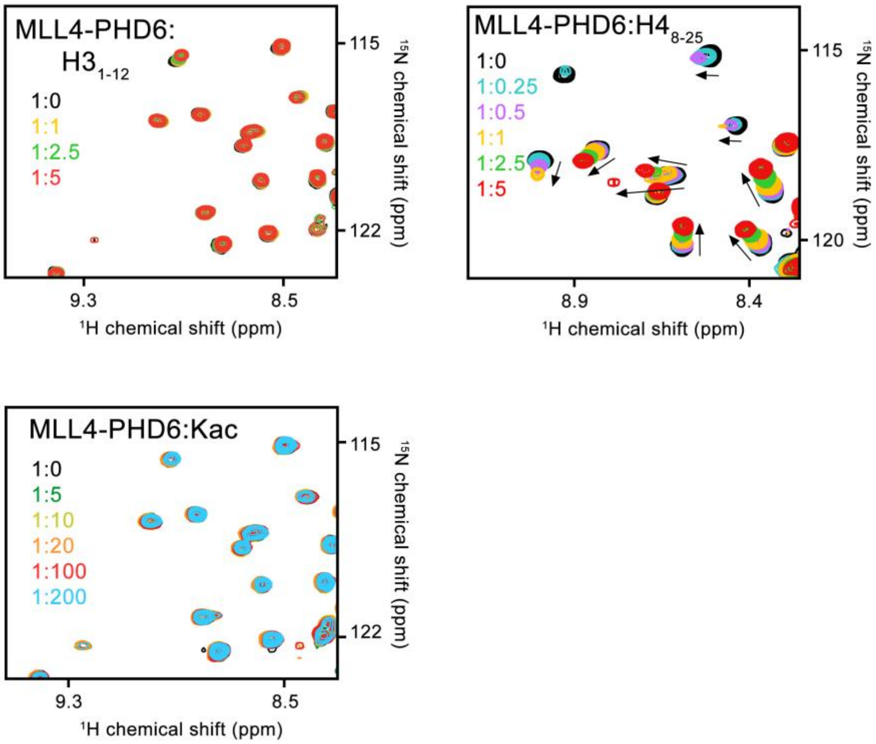

Supplementary Figure 1. MLL4-PHD6 recognizes histone H4 sequence. Superimposed $^{1}H,^{15}N$ HSQC spectra of MLL4-PHD6$_{1503\text{-}1562}$ collected upon titration with indicated histone peptides (top panels) and acetyllysine (bottom panel). Spectra are color coded according to the protein:ligand molar ratio. Related to Figure 1.

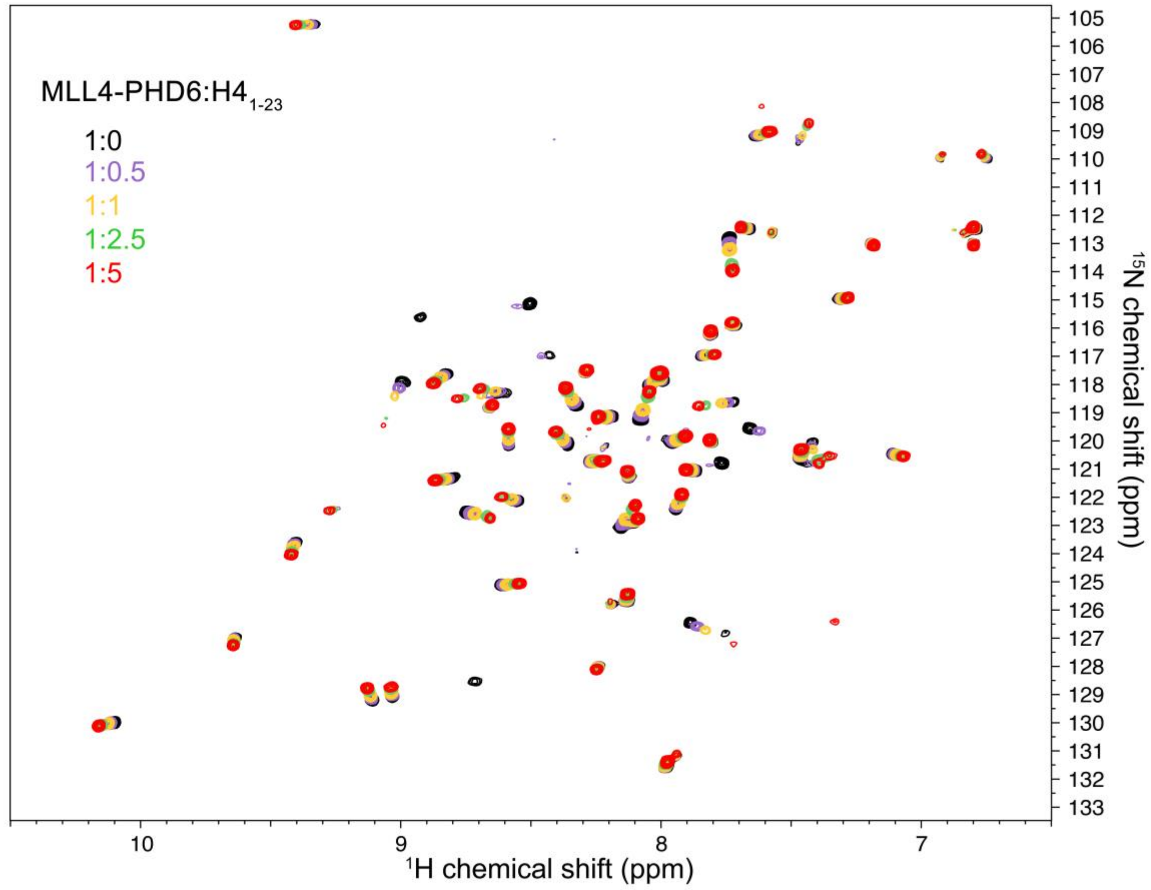

Supplementary Figure 2. MLL4-PHD6 binds to histone H4. Superimposed ^{1}H,^{15}N HSQC spectra of MLL4-PHD6$_{1503-1562}$ collected upon titration with unmodified H4 (1-23) peptide. Spectra are color coded according to the protein:ligand molar ratio. Related to Figure 1.

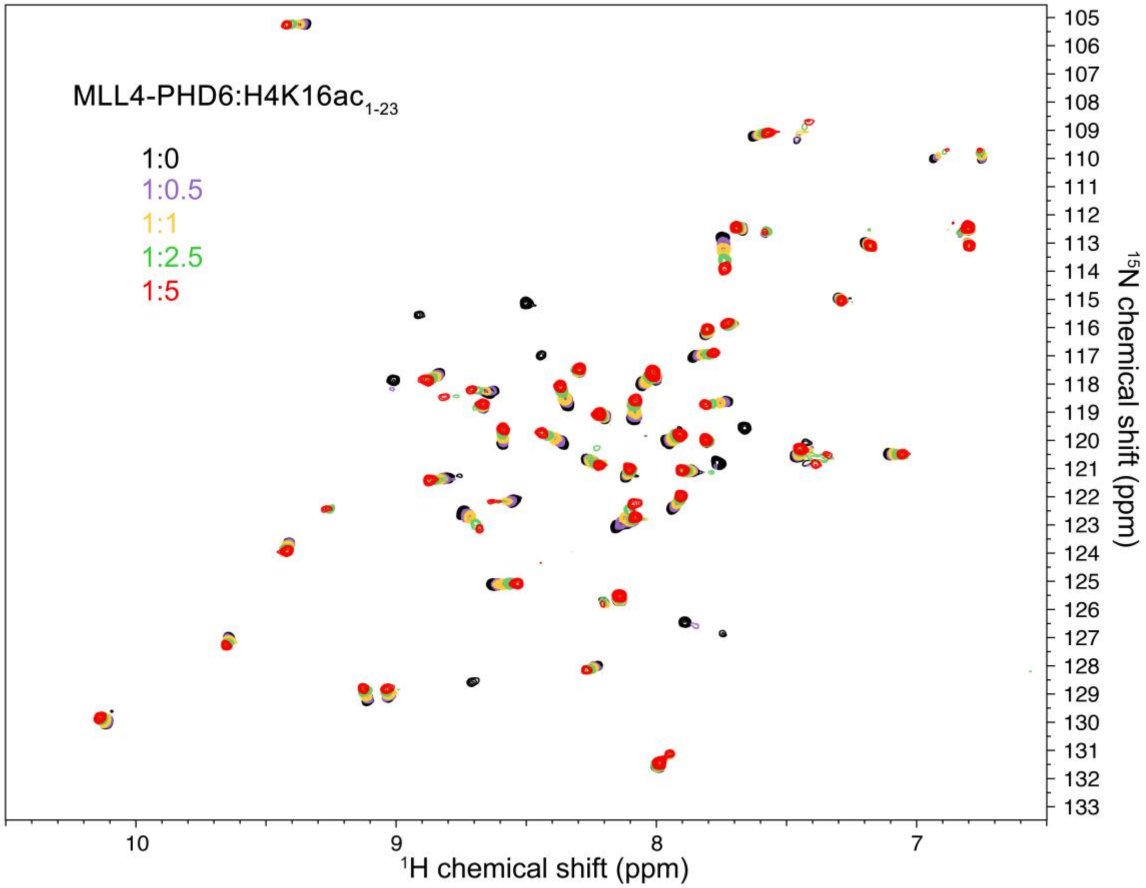

Supplementary Figure 3. MLL4-PHD6 recognizes histone H4K16ac. Superimposed $^{1}H,^{15}N$ HSQC spectra of MLL4-PHD6$_{1503\text{-}1562}$ collected upon titration with H4K16ac (1-23) peptide. Spectra are color coded according to the protein:ligand molar ratio. Related to Figure 1.

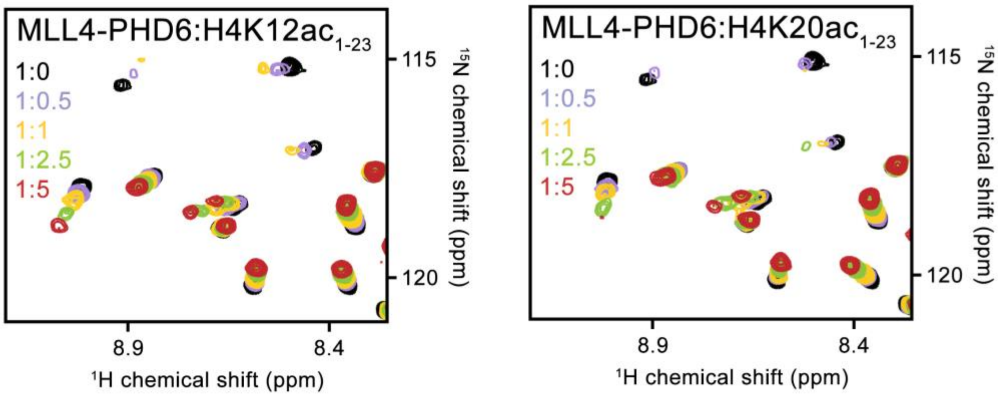

Supplementary Figure 4. MLL4-PHD6 associates with the C-terminal region of H4 tail. Superimposed $^{1}H,^{15}N$ HSQC spectra of MLL4-PHD6$_{1503\text{-}1562}$ collected upon titration with indicated acetylated histone H4 peptides. Spectra are color coded according to the protein:peptide molar ratio. Related to Figure 1.

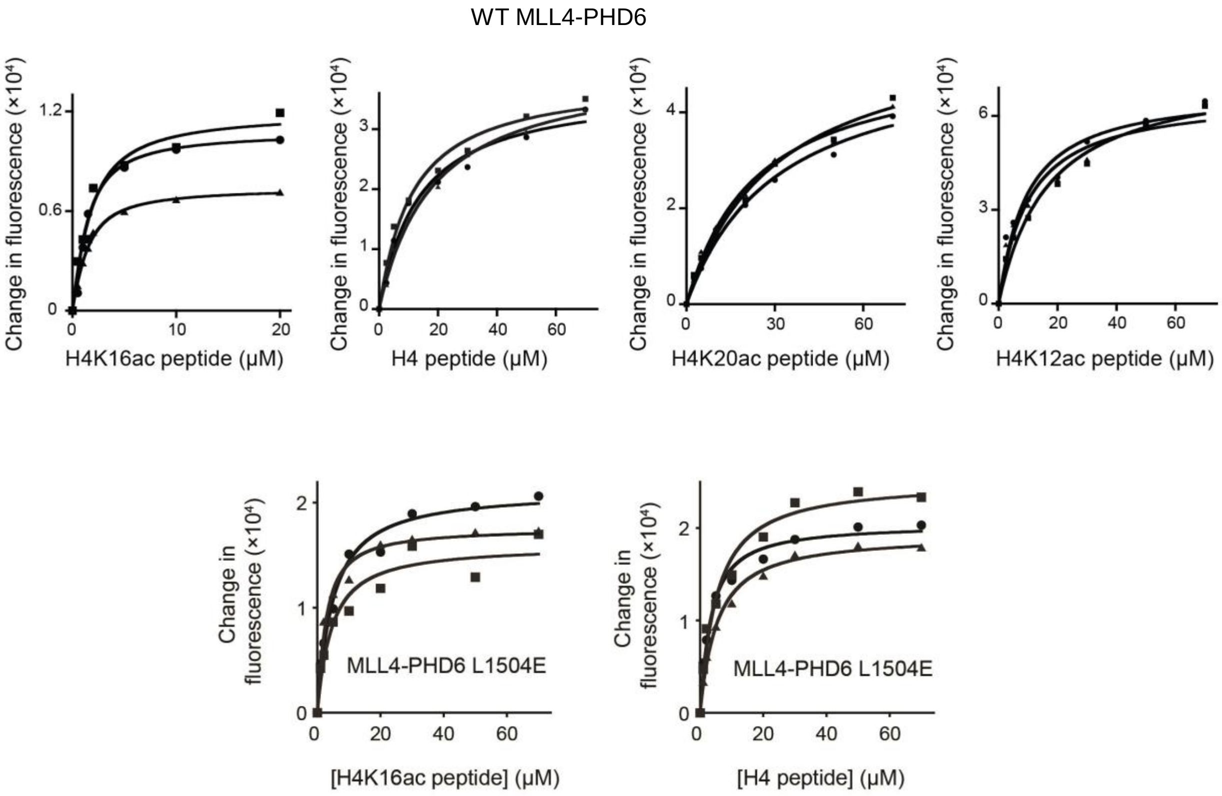

Supplementary Figure 5. Measurements of binding affinities of WT and mutated MLL4-PHD6 to H4 peptides by fluorescence. Binding curves used to determine the K_d values by tryptophan fluorescence for the interactions of (top) WT MLL4-PHD6 and (bottom) L1504E MLL4-PHD6 with the indicated peptides (all aa 1-23 of H4). Related to Figures 1 and 3.

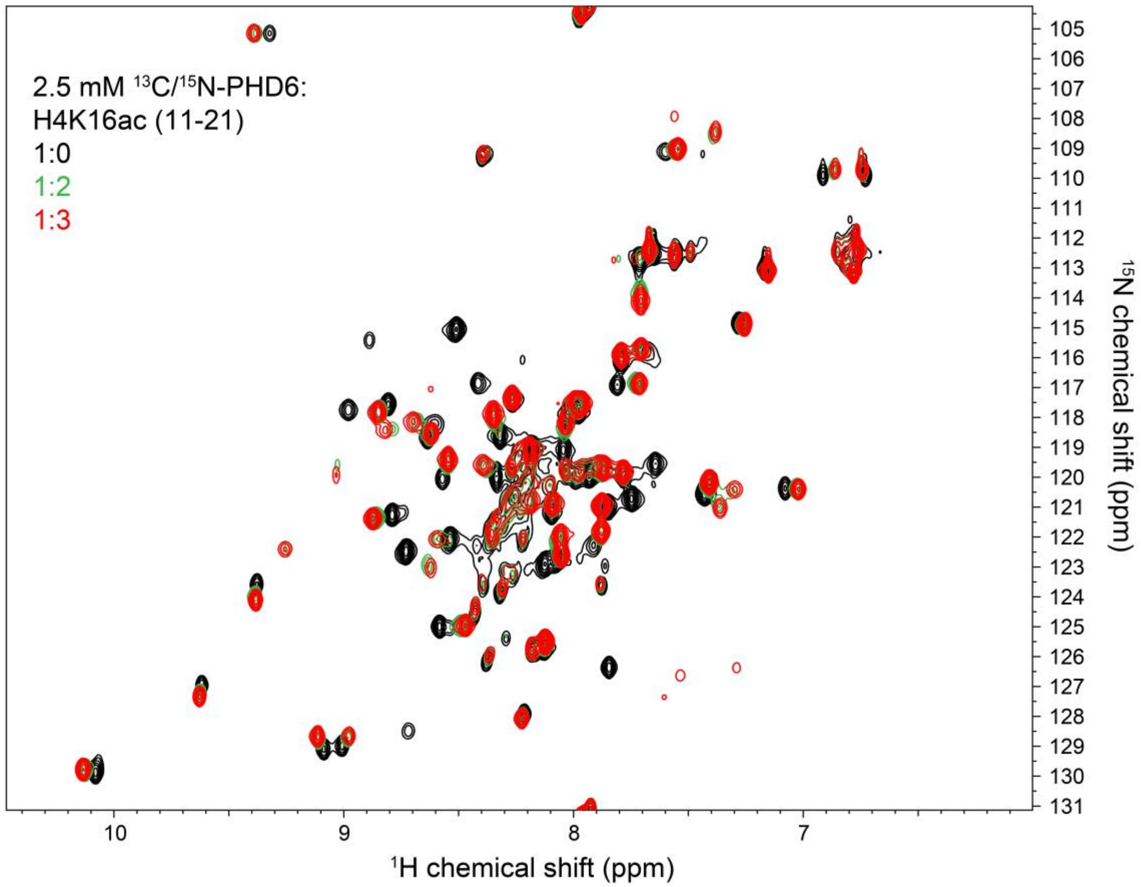

Supplementary Figure 6. Formation of the MLL4-PHD6:H4K16ac complex. Superimposed ^{1}H,^{15}N HSQC spectra of ^{13}C/^{15}N-labeled MLL4-PHD6$_{1503-1562}$ (2.5 mM) collected upon titration with H4K16ac (11-21) peptide. Spectra are color coded according to the protein:ligand molar ratio. Related to Figure 2.

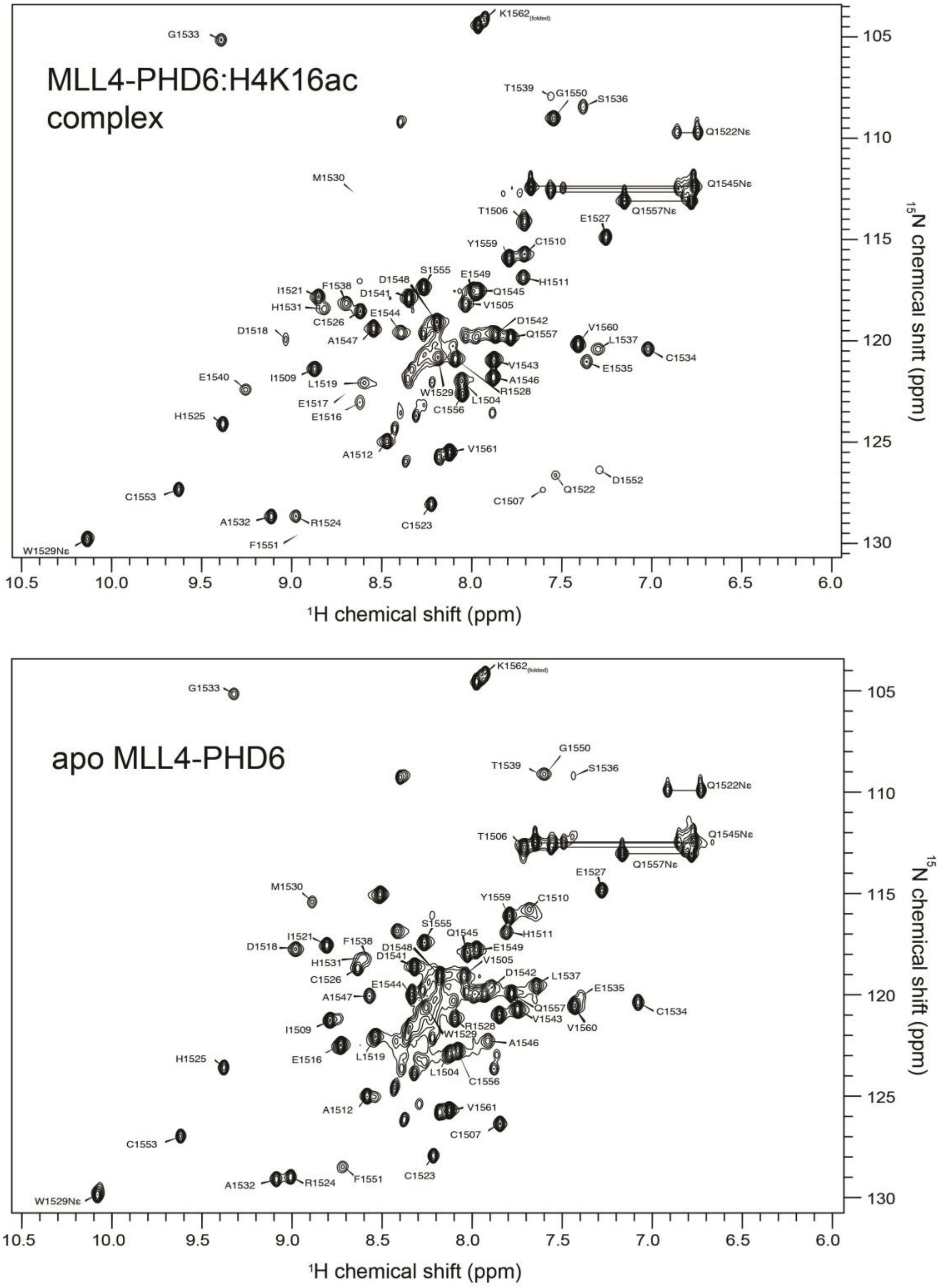

Supplementary Figure 7. Assignments of amide resonances in the MLL4-PHD6:H4K16ac$_{11\text{-}21}$ complex (top) and the apo-state (bottom). ^{1}H,^{15}N HSQC spectra of $^{13}C/^{15}N$-labeled MLL4-PHD6$_{1503\text{-}1562}$ (2.5 mM) in the presence (top) and absence (bottom) of H4K16ac (11-21) peptide (7.5 mM). Related to Figure 2.

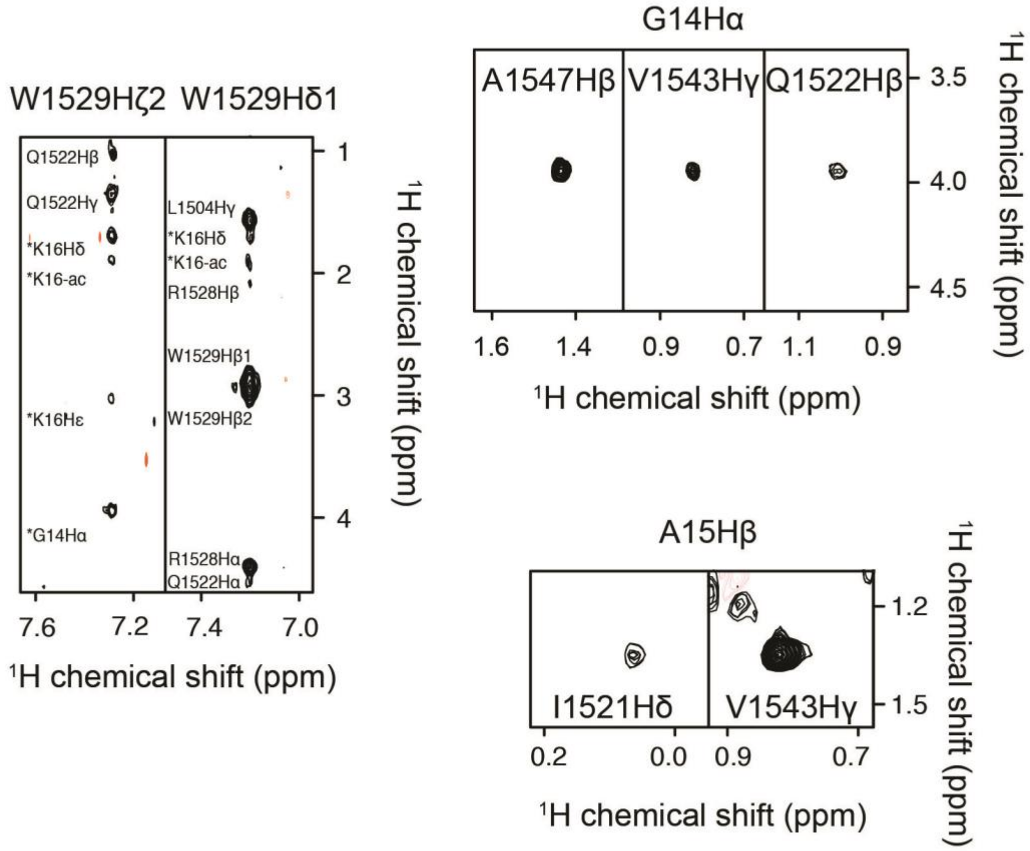

Supplementary Figure 8. Intermolecular NOEs in the MLL4-PHD6:H4K16ac complex. (left) Representative strips from ^{13}C-edited aromatic NOESY experiment using a $^{13}C/^{15}N$-labeled PHD6 sample with a 3-fold excess of H4K16ac peptide. The experiment identifies NOEs to ^{13}C-attached protons in the aromatic sidechains. Both intra- and inter-molecular NOE crosspeaks are observed in the spectra. (right) Representative spectral regions from $^{13}C/^{15}N$-filtered, ^{13}C-edited NOESY experiment using a $^{13}C/^{15}N$-labeled PHD6 sample with a 3-fold excess of H4K16ac peptide. The experiment identifies intermolecular NOEs between protons attached to ^{13}C in PHD6 and protons attached to ^{12}C in H4K16ac. Related to Figure 2.

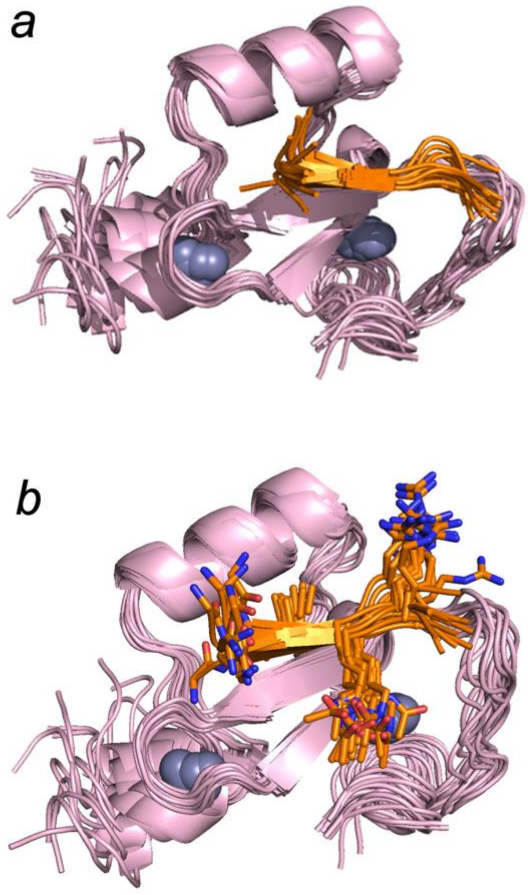

Supplementary Figure 9. Structure of the H4K16ac-bound MLL4-PHD6 domain. Superimposition of 15 final NMR structures of the MLL4-PHD6 bound to the H4K16ac peptide. (a and b) H4K16ac peptide is shown as ribbon. The histone residues Gly13, Gly14, Ala15, Lys16ac, and Arg17 are shown as sticks in (b). Related to Figure 2.

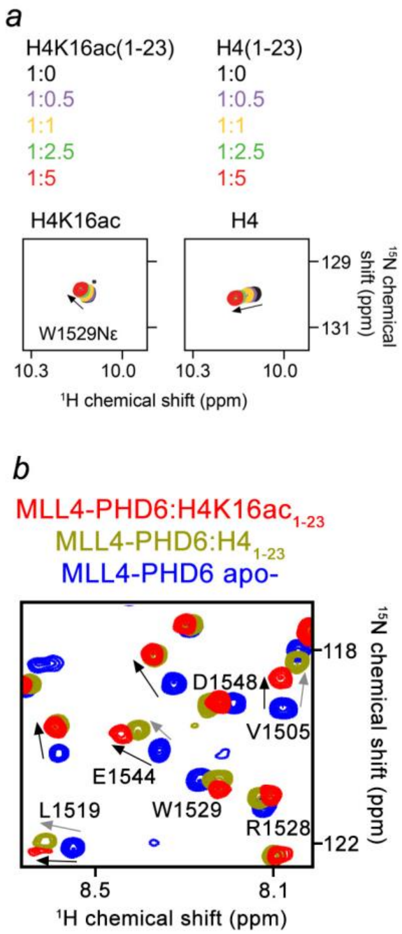

Supplementary Figure 10. Comparison of the interactions of MLL4-PHD6 with H4K16ac and H4 peptides. (a) A zoom-in view of the ^{1}H,^{15}N HSQC spectra shown in Supplementary Figures 2 and 3. (b) Superimposed ^{1}H,^{15}N HSQC spectra of MLL4-PHD6$_{1503-1562}$ in the presence of a 5-fold molar excess of indicated peptides and the apo-state. Related to Figure 3.

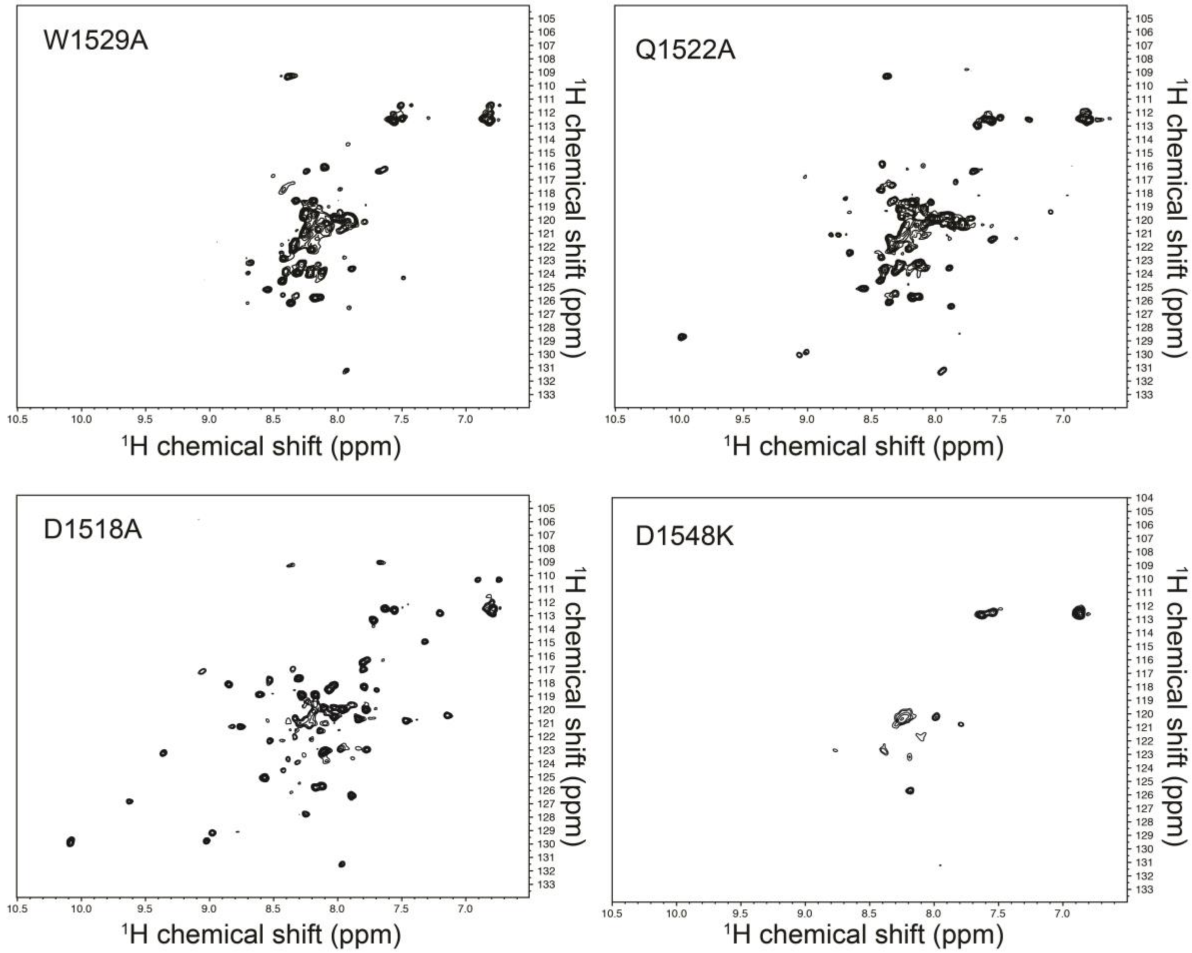

Supplementary Figure 11. Mutations in MLL4-PHD6 domain that disrupt the protein fold and/or stability. $^{1}H,^{15}N$ HSQC spectra of the indicated MLL4-PHD6$_{1503\text{-}1562}$ mutants. Related to Figure 3.

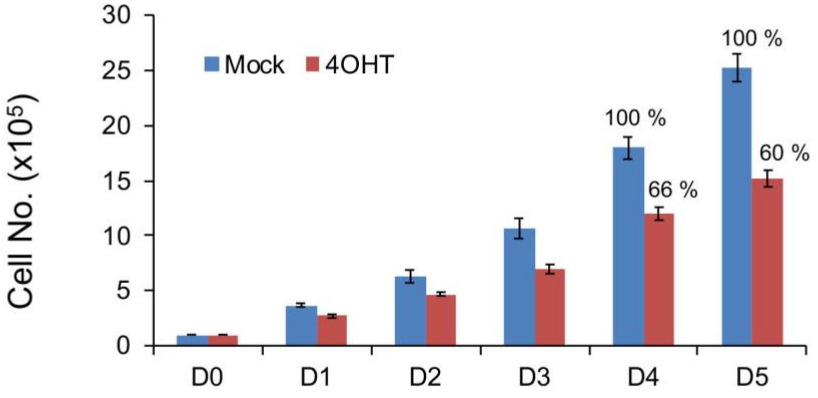

Supplementary Figure 12. ***Mof*** **KO reduces cell growth rates.** *Mof*$^{f/f}$;*Cre-ER* brown preadipocytes were treated with 4OHT to delete *Mof*. Cells were replated for growth curve and counted at indicated time points. The data is presented as mean ± s.d.. Two technical replicates from a single experiment were conducted. Source data are provided as a Source Data file. Related to Figure 4.

49,597 MLL4 target region

GO Term (49,597 MLL4 target regions)	P value
collagen fibril organization	1E-52
cell-substrate junction assembly	9E-47
regulation of RNA stability	5E-36
regulation of reactive oxygen species metabolic process	3E-21
release of cytochrome c from mitochondria	3E-20
protein kinase B signaling cascade	1E-18
protein peptidyl-prolyl isomerization	1E-14
mitotic spindle organization	5E-12

Supplementary Figure 13. MLL4-associated genes in preadipocytes. GO analysis of the genes associated with 49,597 MLL4 binding regions. Related to Figure 5.

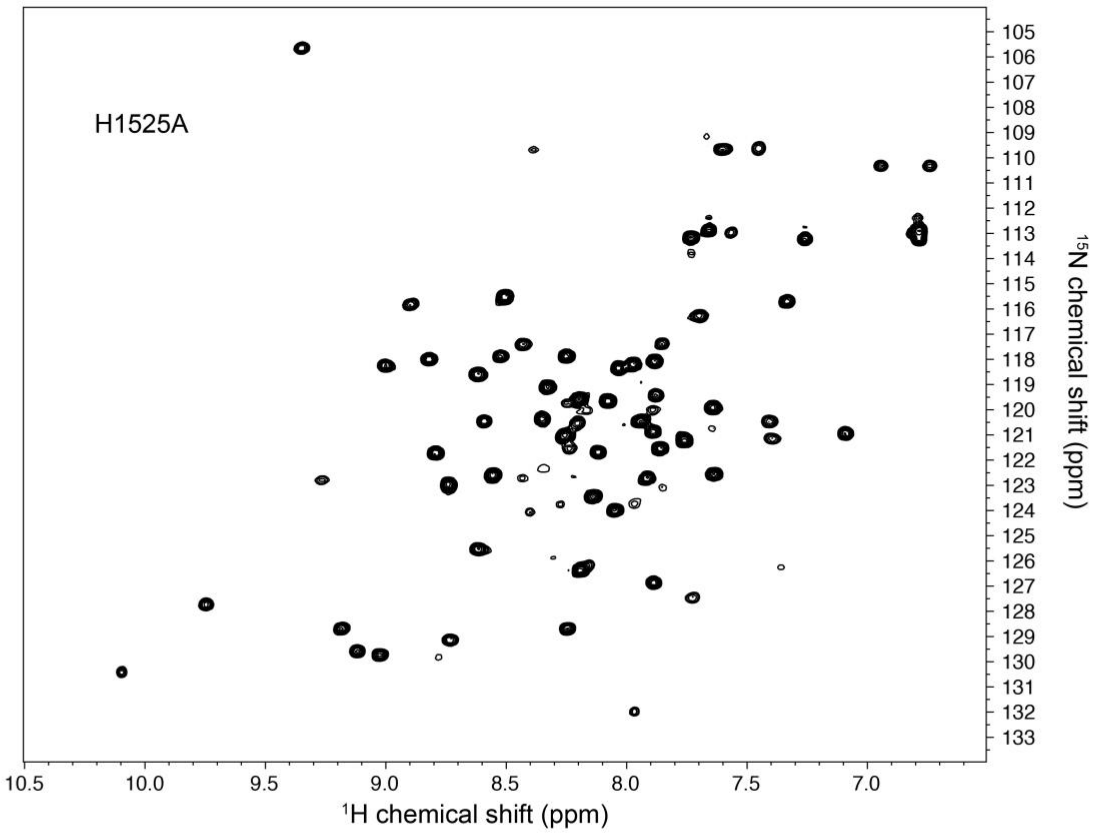

Supplementary Figure 14. H1525 of MLL4-PHD6 is not involved in zinc coordination. $^{1}H,^{15}N$ HSQC spectrum of the H1525A MLL4-PHD6$_{1503\text{-}1562}$ mutant. Related to Figure 2.

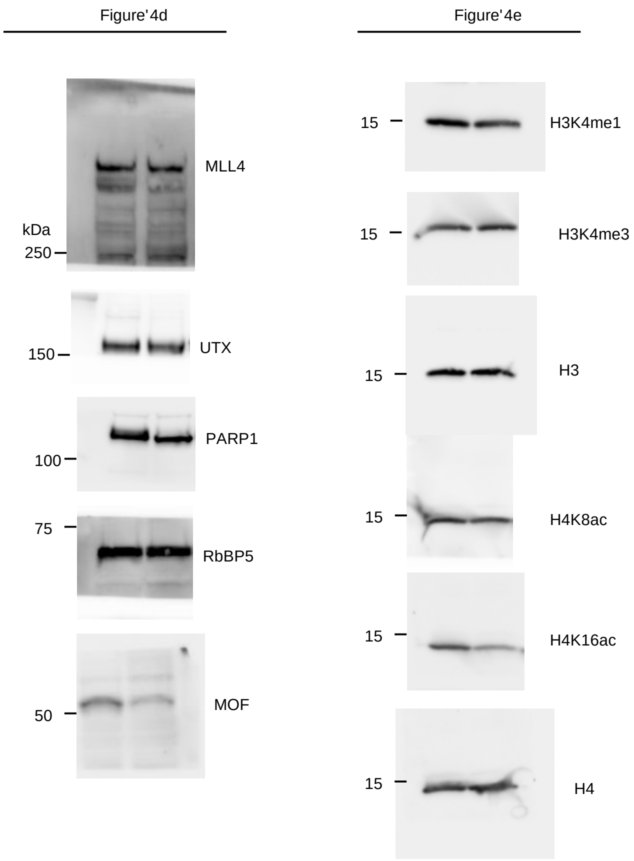

Supplementary Figure 15. Uncropped gels. Related to Figure 4d-e.

Supplementary Table 1. NMR and refinement statistics for the MLL4-PHD6:H4K16ac structure

	MLL4 PHD6-H4K16ac
**NMR distance and dihedral constraints**	
Distance constraints	
Total NOE	1384
Intra-residue	495
Inter-residue	
Sequential ($\|i - j\| = 1$)	309
Medium-range ($\|i - j\| \leq 4$)	417
Long-range ($\|i - j\| \geq 5$)	133
Intermolecular	30
Hydrogen bonds	13
Total dihedral angle restraints	
ϕ	15
ψ	16
**Structure statistics**	
Violations (mean and s.d.)	
Distance constraints (Å)	0.151 ± 0.020
Dihedral angle constraints (º)	0
Max. dihedral angle violation (º)	0
Max. distance constraint violation (Å)	0.205
Deviations from idealized geometry	
Bond lengths (Å)	0.0113 ± 0.0003
Bond angles (º)	2.531 ± 0.039
Average pairwise r.m.s. deviation** (Å)	
Heavy	1.387 ± 0.243
Backbone	0.968 ± 0.229

**Pairwise r.m.s. deviation to lowest energy structure was calculated among 15 refined structures. Residues 1504-1558 of MLL4 and 14-17 of H4 were used for calculation.

Supplementary Table 2. List of primers used in this study.

PHD6_NdeI_f	GGATCTCATATGAGCCTGGTGACCTG
PHD6_BamHI_r	GAATTGGGATCCttaCTTTACCACGTAG
GA_universal_f	ggACAAGTTTGTACAAAAAAGCAGGCTCGGAGAATTTGTATTTTCAG
TEV_H4_11_f	GAGAATTTGTATTTTCAGggcaagggtggcgcgaaac
H4_K12A_f	GAGAATTTGTATTTTCAGGGCgctGGTGGCGCGAAACGCCATC
H4_K12A_r	GATGGCGTTTCGCGCCACCagcGCCCTGAAAATACAAATTCTC
H4_K16AR17A_f	CAGGGCAAGGGTGGCGCGgctgcCCATCGTAAAGTGCTGCGTG
H4_K16AR17A_r	CACGCAGCACTTTACGATGGgcagcCGCGCCACCCTTGCCCTG
H4_R23A_f	GTAAAGTGCTGgcTGGTGGAGGCGGAGGTGGAGGCAGCCTG
H4_R23A_r	CGCCTCCACCAgcCAGCACTTTACGATGGCGTTTCGCGCCAC
PHD6_attB2_r	CAAGAAAGCTGGGTCTTACACGTAGGGCTGGCAG
E1540K_f	TGAGAGCCTCTTCACAAAGGACGATGTGGAGCAG
E1540K_r	CTGCTCCACATCGTCTTCTGTGAAGAGGCTCTCA
E1544_f	CACAGAGGACGATGTGAAGCAGGCAGCCGATGAA
E1544_r	TTCATCGGCTGCCTGCTTCACATCGTCCTCTGTG
H1525A_f	CTAATCCAGTGCCGCgcCTGTGAACGGTGGATG
H1525A_r	CATCCACCGTTCACAGgcGCGGCACTGGATTAG
W1529A_f	CACTGTGAACGGgctATGCATGCAGGCTGTGAGAG
W1529A_r	CTGCATGCATagcCCGTTCACAGTGGCGGCAC
Q1522A_f	GAGGACCTACTAATCgctTGCCGCCACTGTGAACG
Q1522A_r	CGTTCACAGTGGCGGCAagcGATTAGTAGGTCCTC
D1548K_f	GATGTGGAGCAGGCAGCCAAGGAAGGCTTTGACTG
D1548K_r	CAGTCAAAGCCTTCCTTGGCTGCCTGCTCCACATC
Mof_fw	TGAGATCAACCATGTGCAGAAG
Mof_rev	AGGTGAGAAATACCAGGCATC
Jmjd3_fw	TGAAGAACGTCAAGTCCATTGTG
Jmjd3_rev	TCCCGCTGTACCTGACAGT
