# Supplementary material for: Selective binding of the PHD6 finger of MLL4 to histone H4K16ac links MLL4 and MOF
Source: Nat Commun. 2019 May 24;10:2314. doi: 10.1038/s41467-019-10324-8 (PMC6534582; doi:10.1038/s41467-019-10324-8)
Supplement: Supplementary file 3 — Reporting Summary [file 41467_2019_10324_MOESM3_ESM.pdf]

nature research

Corresponding author(s): Tatiana Kutateladze, Kai Ge

Reporting Summary

Nature Research wishes to improve the reproducibility of the work that we publish. This form provides structure for consistency and transparency in reporting. For further information on Nature Research policies, see Authors & Referees and the Editorial Policy Checklist.

Statistical parameters

When statistical analyses are reported, confirm that the following items are present in the relevant location (e.g. figure legend, table legend, main text, or Methods section).

n/a	Confirmed	
☐	☒	The exact sample size (*n*) for each experimental group/condition, given as a discrete number and unit of measurement
☐	☒	An indication of whether measurements were taken from distinct samples or whether the same sample was measured repeatedly
☒	☐	The statistical test(s) used AND whether they are one- or two-sided<br>*Only common tests should be described solely by name; describe more complex techniques in the Methods section.*
☒	☐	A description of all covariates tested
☒	☐	A description of any assumptions or corrections, such as tests of normality and adjustment for multiple comparisons
☒	☐	A full description of the statistics including central tendency (e.g. means) or other basic estimates (e.g. regression coefficient) AND variation (e.g. standard deviation) or associated estimates of uncertainty (e.g. confidence intervals)
☒	☐	For null hypothesis testing, the test statistic (e.g. *F*, *t*, *r*) with confidence intervals, effect sizes, degrees of freedom and *P* value noted<br>*Give P values as exact values whenever suitable.*
☒	☐	For Bayesian analysis, information on the choice of priors and Markov chain Monte Carlo settings
☒	☐	For hierarchical and complex designs, identification of the appropriate level for tests and full reporting of outcomes
☒	☐	Estimates of effect sizes (e.g. Cohen's *d*, Pearson's *r*), indicating how they were calculated
☐	☒	Clearly defined error bars<br>*State explicitly what error bars represent (e.g. SD, SE, CI)*

Our web collection on statistics for biologists may be useful.

Software and code

Policy information about availability of computer code

Data collection	NMR experiments were carried out at 298K on Varian INOVA 600 and 900 MHz spectrometers at the UC Denver NMR Core facility
Data analysis	CcpNmr Suite, Amber16, and other software listed in the Method section. Software for ChIP-seq analysis include bowtie2; SICER algorithm, GREAT v3, SeqPos listed in Method section.

For manuscripts utilizing custom algorithms or software that are central to the research but not yet described in published literature, software must be made available to editors/reviewers upon request. We strongly encourage code deposition in a community repository (e.g. GitHub). See the Nature Research guidelines for submitting code & software for further information.

Data

Policy information about availability of data

All manuscripts must include a data availability statement. This statement should provide the following information, where applicable:

- Accession codes, unique identifiers, or web links for publicly available datasets
- A list of figures that have associated raw data
- A description of any restrictions on data availability

The atomic coordinates and NMR assignments of MLL4-PHD6 in complex with H4K16ac peptide have been deposited in the Protein Data Bank under the accession

codes 6O7G. The ChIP-seq data is submitted to Gene Expression Omnibus under the accession number is GSE130091. Other data are available from the corresponding author upon reasonable request.

Field-specific reporting

Please select the best fit for your research. If you are not sure, read the appropriate sections before making your selection.

☒ Life sciences ☐ Behavioural & social sciences ☐ Ecological, evolutionary & environmental sciences

For a reference copy of the document with all sections, see nature.com/authors/policies/ReportingSummary-flat.pdf

Life sciences study design

All studies must disclose on these points even when the disclosure is negative.

Sample size	present in relevant figure legends
Data exclusions	no data exclusions
Replication	present in relevant figure legends
Randomization	no randomization
Blinding	no blinding

Reporting for specific materials, systems and methods

Materials & experimental systems

n/a	Involved in the study	
☒	☐	Unique biological materials
☐	☒	Antibodies
☐	☒	Eukaryotic cell lines
☒	☐	Palaeontology
☒	☐	Animals and other organisms
☒	☐	Human research participants

Methods

n/a	Involved in the study	
☐	☒	ChIP-seq
☒	☐	Flow cytometry
☒	☐	MRI-based neuroimaging

Antibodies

Antibodies used	Antibodies used for ChIP are described in the ChIP-seq report section. Antibodies used for other assays: anti-MLL4#336 and anti-UTX37. Anti-MOF (A300-992A) and anti-RbBP5 (A300-109A) were from Bethyl Laboratories. Anti-H3 (ab1791), anti-H3K4me1 (ab8895), and anti-H4 (ab7311) were from Abcam. Anti-H3K4me3 (07-473), anti-H4K8ac (07-328), and anti-H4K16ac (07-329) were from Millipore. Anti-PARP1 (556362) were from BD bioscience. Anti-MYC (sc40) was from Santa Cruz.
Validation	All antibodies validation are available on the manufacturers' websites.

Eukaryotic cell lines

Policy information about cell lines

Cell line source(s)	Mouse strains and cell lines are generated and listed in the method section.
Authentication	*Describe the authentication procedures for each cell line used OR declare that none of the cell lines used were authenticated.*
Mycoplasma contamination	*Confirm that all cell lines tested negative for mycoplasma contamination OR describe the results of the testing for mycoplasma contamination OR declare that the cell lines were not tested for mycoplasma contamination.*
Commonly misidentified lines (See ICLAC register)	*Name any commonly misidentified cell lines used in the study and provide a rationale for their use.*

ChIP-seq

Data deposition

☒ Confirm that both raw and final processed data have been deposited in a public database such as GEO.

☒ Confirm that you have deposited or provided access to graph files (e.g. BED files) for the called peaks.

Data access links<br>*May remain private before publication.*	https://www.ncbi.nlm.nih.gov/geo/query/acc.cgi?acc=GSE130091
Files in database submission	Mof_CreER_Vec_D-3_Mock_MLL4.fastq.gz<br>Mof_CreER_WT_rMof_D-3_4OHT_MLL4.fastq.gz<br>Mof_CreER_Vec_D-3_4OHT_Input.fastq.gz<br>Mof_CreER_K274R_rMof_D-3_4OHT_Input.fastq.gz<br>Mof_CreER_Vec_D-3_4OHT_H4K16ac.fastq.gz<br>Mof_CreER_K274R_rMof_D-3_4OHT_H4K16ac.fastq.gz<br>Mof_CreER_Vec_D-3_4OHT_MLL4.fastq.gz<br>Mof_CreER_WT_rMof_D-3_4OHT_Input.fastq.gz<br>Mof_CreER_K274R_rMof_D-3_4OHT_MLL4.fastq.gz<br>Mof_CreER_Vec_D-3_Mock_H4K16ac.fastq.gz<br>Mof_CreER_WT_rMof_D-3_4OHT_H4K16ac.fastq.gz<br>Mof_CreER_Vec_D-3_Mock_Input.fastq.gz<br>Mof_CreER_Vec_D-3_Mock_MLL4_sorted-W50-G50-FDR1E-10-islandfiltered-normalized.wig<br>Mof_CreER_WT_rMof_D-3_4OHT_MLL4_sorted-W50-G50-FDR1E-10-islandfiltered-normalized.wig<br>Mof_CreER_K274R_rMof_D-3_4OHT_H4K16ac_sorted-W200-G200-FDR1E-3-islandfiltered-normalized.wig<br>Mof_CreER_Vec_D-3_4OHT_H4K16ac_sorted-W200-G200-FDR1E-3-islandfiltered-normalized.wig<br>Mof_CreER_Vec_D-3_4OHT_MLL4_sorted-W50-G50-FDR1E-10-islandfiltered-normalized.wig<br>Mof_CreER_WT_rMof_D-3_4OHT_H4K16ac_sorted-W200-G200-FDR1E-3-islandfiltered-normalized.wig<br>Mof_CreER_K274R_rMof_D-3_4OHT_MLL4_sorted-W50-G50-FDR1E-10-islandfiltered-normalized.wig<br>Mof_CreER_Vec_D-3_Mock_H4K16ac_sorted-W200-G200-FDR1E-3-islandfiltered-normalized.wig
Genome browser session<br>(e.g. UCSC)	no longer applicable

Methodology

Replicates	ChIP-Seq data are from single experiment.
Sequencing depth	Sequencing was done at 50bp, single-ended.
Antibodies	anti-H4K16ac (Millipore, 07-329)<br>anti-MLL4 (home-made)
Peak calling parameters	For MLL4 peak calling, a window size of 50 bp and a gap size of 50 bp was used. For H4K16ac, a window size of 200 bp and a gap size of 200 bp was used.
Data quality	For MLL4, peaks with FDR<1E-10 were included in the data analysis. For H4K16ac, FDR<1E-3 were included.
Software	Software for ChIP-seq analysis include bowtie2; SICER algorithm, GREAT v3, SeqPos listed in Method section.
